# Like Frying Multiple Eggs in One Pan: a Qualitative Study Exploring the Understanding of Inter-speciality Training in Cancer Care

**DOI:** 10.1007/s13187-023-02285-w

**Published:** 2023-04-03

**Authors:** W. McInally, K. Benstead, A. Brandl, N. Dodlek, J. De Munter, C. Gasparotto, J. Grau-Eriksen, R. G. Kelly, C. Lecoq, N. O’Higgins, K. Oliver, M. Popovics, I. Rollo, V. Sulosaari, Celia Diez de los Rios de la Serna

**Affiliations:** 1 The Open University, Milton Keynes, UK; 2 Gloucestershire Hospitals NHS Foundation Trust, Gloucester, UK; 3 Charite Berlin, Berlin, Germany; 4 Cyprus University of Technology, Limassol, Cyprus; 5 Ghent University Hospital, Brussels, Belgium; 6 European Society for Radiotherapy and Oncology, Brussels, Belgium; 7 Aarhus University Hospital, Aarhus, Denmark; 8 European Oncology Nursing Society, Brussels, Belgium; 9 European Society of Surgical Oncology, Brussels, Belgium; 10 University College Dublin, Dublin, Ireland; 11 European Cancer Organisation, Brussels, Belgium; 12 Turku University of Applied Sciences, Turku, Finland; 13 University of Barcelona, Barcelona, Spain

**Keywords:** Healthcare professionals, Experiences, Cancer, Care, Education, Inter-specialty training

## Abstract

H igh-quality cancer care is a key priority worldwide. Caring for people affected by cancer requires a range of specific knowledge, skills and experience to deliver the complex care regimens both within the hospital and within the community environment. In June 2022, the European Cancer Organisation along with 33 European cancer societies began working together to develop a curriculum for inter-speciality training for healthcare professionals across Europe. As part of the project, this research consisted of a qualitative survey distributed to the European Union societies via email. The aim of this paper is to disseminate the qualitative findings from healthcare professionals across Europe. Questionnaires were sent out to a convenience sample of 219 healthcare professionals and patient advocates with a response rate of 55% (*n* = 115). The findings identified that there were four key themes: ‘What is inter-speciality training?’, ‘Barriers and challenges’, ‘Support throughout the cancer journey’ and ‘New ways of working’. These results are part of a larger needs analysis and scoping review to inform the development of a core competency framework which will be part of an inter-speciality curriculum for specialist cancer doctors, nurses and other healthcare professionals across Europe. Healthcare professionals will be able to access education and training through the virtual learning environment and workshops and by clinical rotations to other specialties.

## Introduction

Cancer is a key priority worldwide, and caring for people affected by cancer requires a range of specific knowledge, skills and experience in order to deliver the complex care regimes both within the hospital and within the community environment [1]. The European Union [2] states that cancer is one of the top priorities for all people affected by cancer and healthcare professionals (HCPs) must be suitably prepared and equipped. This statement is reinforced by the European Code of Cancer Practice [3] (p. 35) and is one of its ten key overarching rights, “You have a right to receive care from a specialised multidisciplinary team, ideally as part of a cancer care network”. It is also essential to understand what is necessary within the cancer care pathway for HCPs to work together holistically to ensure this level of care is provided for all people affected by cancer and their families [4, 5].

Europe’s Beating Cancer Plan [2] and one of the five missions included in the new Horizon Europe programme [6] identified that cancer is diagnosed in 3.5 million people each year across Europe. Education and training of all HCPs to cope with this number are of the utmost importance. Competence to enable clinical, radiation and medical oncologists; cancer surgeons; radiologists; and cancer nurses to work more effectively together to deliver better care to patients is being developed as part of the INTERACT-EUROPE programme (https://www.europeancancer.org/eu-projects/impact/interact-europe). This study contributes to that process together with a scoping review of the literature and a learning needs analysis.

Modern healthcare organisations recognise the importance of inter-professional learning (IPL), training and collaboration. These components form an additional aspect of each professional group’s specific competence, where the cancer care teams are equipped to meet the complex needs in modern healthcare [2, 3, 5, 6]. There is an increasing emphasis on education and training future HCPs to learn with, from and about each other with the goal of improving health outcomes through more effective inter-professional collaboration [7].

IPL and inter-speciality training (IST) provide the opportunity for HCPs from different specialties and professions to learn and work together to provide the patient and their family with the highest-quality care. The World Health Organization [7] has, since the 1980s, promoted IPL in healthcare as an important method to increase the quality of patient and family care. There is evidence supporting team learning, working and development [8, 9]. Despite this, few healthcare higher education institutions provide IPL at postgraduate level to any great extent. It is notable that, with few exceptions, education and training programmes for cancer nursing, medical specialists in cancer care (medical oncologists, radiation oncologists and cancer surgeons/surgical oncologists) are arranged and conducted independently of each other with little or no IPL or IST through education or training.

Although from the beginning, it was clear that the HCPs and patient advocates all had a different knowledge and understanding of what IST is. For the purposes of this, it was agreed on a definition for IST as part of the programme. “Inter-specialty training in oncology occurs when two or more professions collaborate by learning and interacting with each other during training in order to provide high quality cancer care”.

INTERACT-EUROPE, led by European Cancer Organisation (ECO), is developing an inter-specialty oncology curriculum. This paper will report on the qualitative aspect of the learning needs analysis that was used to influence the development of the competencies for the curriculum. This process also involved a quantitative survey investigating the value placed on possible competencies by professional and patient advocate groups. The elements of the curriculum including the entrance requirements of trainees, qualifications of trainers, design of an online programme, methods of assessment, communication of the details of the curriculum to a wide European audience and evaluation of the programme are being developed by a combination of online surveys and face-to-face consultations.

### Research Aims and Objectives

The aims of this qualitative survey were to.

- Gather the participants’ experiences of IST
- Identify the challenges and barriers in delivery
- Determine why IST was considered necessary to influence the development of the competency framework for the INTERACT-EUROPE programme.

## Methods

An online SurveyMonkey questionnaire was created by a team comprising representatives of clinical oncology, medical oncology, radiation oncology, nursing surgeons and patient advocates. This included a short survey investigating the perceived value of a curriculum for the professions and specialties involved in the care of the patient with cancer. A survey investigates the agreement with the value of a series of proposed competence when participants were asked to score them using the question, “What is it valuable for trainees to learn in order to work more effectively with different specialties and professions to deliver better care and to provide psychosocial and nutritional support”. The final part of the survey was eight qualitative open-ended questions which were distributed to the HCPs (see Table 1).

**Table 1:** Questions

1. What is your understanding of inter-speciality training (IST)?
2. Why is IST necessary in caring for people affected by cancer and their family?
3. What barriers/challenges are there to accessing IST and education within your role as a healthcare professional?
4. Are there any barriers/challenges to working in an IST context?
5. Have you experienced IST improving the cancer journey for patients and their families and/or for you as a healthcare professional?
6. Describe a time when you were particularly proud of your professional healthcare team and why?
7. What was your role in that situation?
8. In your opinion, what are the three most important learnings that trainees should take away from an IST curriculum?
9. Do you have any further comments you would like to add?

Questions 6 and 7 were analysed together

### Data Collection

The questionnaire was distributed by email through SurveyMonkey to 33 EU societies. These societies were approached by the consortium member societies and the patient advocates through ECO.

### Sample

**Fig. 1:**
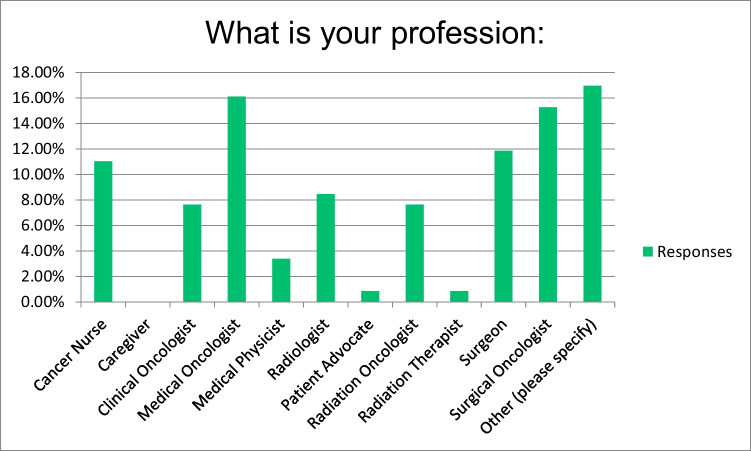
Participants of the study

Convenience samples were sent to 219 HCPs across Europe, with 115 (55%) responding to the qualitative survey. The sample represented cancer nurses, clinical and medical oncologists, patient advocate, radiation oncologists’ cancer surgeons and others (although not specified) Fig. 1 provides a breakdown of the participants.

### Data Analysis

Data analysis is central to credible qualitative research [10] and important to gather a rich description of often complex phenomena giving the narrative voice. Indeed, the qualitative researcher is often described as the research instrument to understand, describe and interpret experiences and perceptions is a key to uncovering meaning in particular circumstances and contexts [11].

The framework devised by Braun and Clarke [12] was applied in a systematic manner in order to describe and explain the process of analysis within the context of learning and teaching research. This process was carried out by two researchers. The transcripts were coded inductively, read and re-read to become familiar with the data. Initial codes were generated from the data and subsequently organised and re-organised, searching for themes and sub-themes. Themes were reviewed through a deductive re-analysis process by the wider project team; themes which lacked sufficient data were discarded. The final themes were named, defined and written up. Colour coding was used through Microsoft Word, along with felt tip pens and paper, after which themes began to emerge for discussion. This shift from coding should maintain complexity and depth, which was created through exploratory coding while also reducing the amount of data (see Table 2). Reflective journaling was also carried out to ensure critical reflection on the process of data collection and analysis.

**Table 2:**
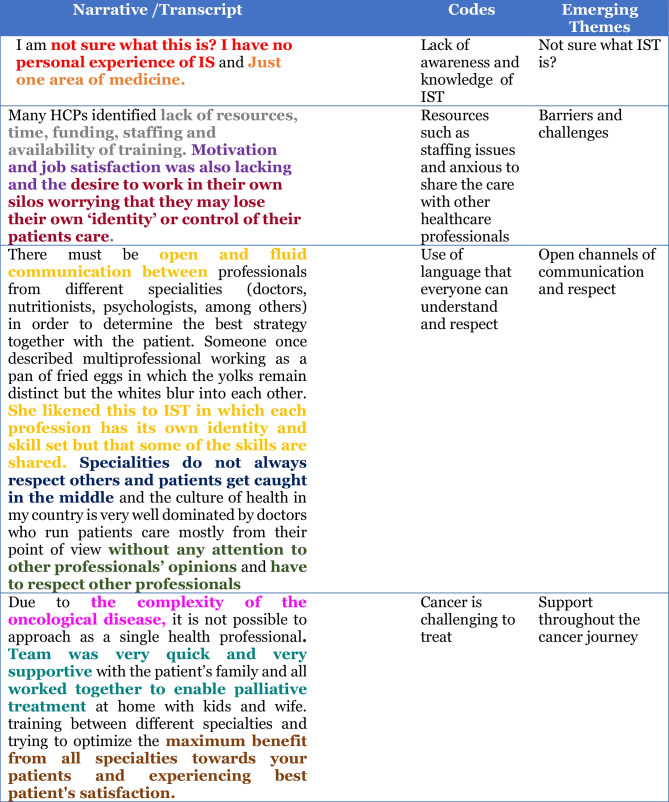
JCE

### Ethical

The project consortium with the coordinator sent all documentation for review by the internal team. All participants received information explaining the purpose of the research and that all responses were anonymised and stored according to General Data Protection Regulation (GDPR) rights [13]. The GDPR balances the right for privacy with the free exchange of data that is important for progress throughout the EU. Completion and submission of the responses was regarded as the participants’ consent to take part in the survey. Access to the questionnaire data was restricted to the researchers who carried out the qualitative study.

### Findings

#### Theme 1: What Is IST?

This theme emerged which clearly revealed that the participants all had different experiences, as one young medical physicist expressed “I don’t know. I would guess for medical doctors to learn about different specialty”. Many HCPs, especially the young medical trainees, did not know what IST is. They were unable to identify the delivery of truly integrated cancer care, bringing together specialist cancer care providers and primary care professionals, whose roles are recognised as crucial. Nevertheless, most did have knowledge of IST and believed this was the best way to care for patients and their families affected by cancer. “When approaching a patient with cancer, it is essential that each professional in their area accompany the patient and family on their journey” (cancer nurse).

#### Theme 2: Barriers and Challenges

This theme illustrates that many HCPs identified a lack of resources, time, funding, staffing and availability of training as barriers to implementation of IST. Lack of motivation and poor job satisfaction were also considered challenges to a new type of training. Some respondents thought that they would prefer to work in their own silos and were concerned that IST might result in loss of their own ‘identity’ or control of their patients care. As one medical oncologist describes, “We don’t have this training in my country. Firstly, the health system must be changed. Unfortunately, cancer care is not provided by public health. As a healthcare professional, I don’t have a lot of time and access to IST”. Other obstacles included the lack of opportunity for IST and that because most EU countries have their own ways of working in accordance with established policy and procedures.

#### Theme 3: Support Throughout the Cancer Journey

Poor communication is a major issue across the multidisciplinary team (MDT), many respondents stating that this is a severe deficiency. Participants reflected on their own experiences of caring for people affected by cancer and how important language and communication was within the multidisciplinary team and with the patient and family. This was vital to ensure a true understanding of the patient journey and, ultimately, the cancer journey. This is captured in the narrative which suggests that this is an area that the project team needs to consider in the development of the curriculum. As one clinical oncologist suggested, “There must be open and fluid communication between professionals from different specialities (doctors, nutritionists, psychologists, among others) to determine the best strategy together with the patient. They further described multiprofessional working as a pan of fried eggs in which the yolks remain distinct, but the whites blur into each other”. This derivative quotation illustrates the distinction between working independently and working together as a team.

Open channels of communication were also seen as important between HCPs and especially for the patient and their family. In addition, being mindful of the terminology used in the content within the curriculum is also important to ensure all HCPs understand the written document in order to improve communication and avoid confusion.

#### Theme 4: New Ways of Working

IST was suggested as working together rather than apart as most HCPs tend to do within their countries. This theme highlights team working for the holistic care of the patient and remains central to the way in which education and training needs to be considered. Most participants agreed on this way of working as indicated by statements such as “we haven’t been good at working in this way but we need to be” (radiologist) and “colleagues in palliative care are good at this and we need to learn from them” (medical oncologist).

The desire for closer professional collaborative arrangements was expressed by all participants irrespective of their specialty. For the most part, they wished to have rotations as part of the IST curriculum: “When trainees from a speciality have rotations in different but linked specialities in order to have a general understanding of their principles and there is no other way” (clinical oncologist). Most participants highlighted those new ways of working are essential.

## Discussion

**Fig. 2:**
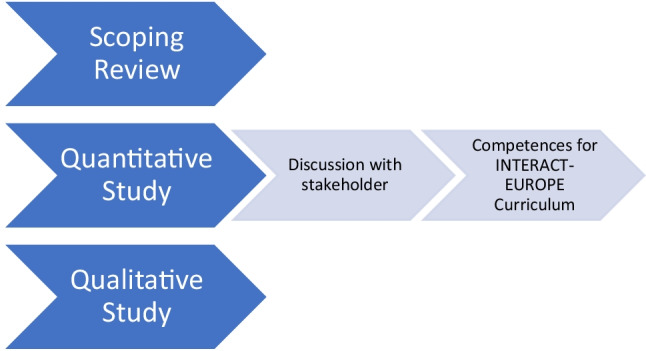
Conceptual model of learning needs

These findings suggest that while most of the participants recognised the value of IPL, there was evidence of confusion, challenges and barriers around IST with many having no experience of this type of learning which is a relatively new and innovative concept. The study highlighted the lack of a universally understood/consensus definition of IST amongst those surveyed. Its importance is emphasised in the development of a core competency framework in which an explicit and clear explanation of IST is outlined. In planning a curriculum, there are many questions to be answered [14]. Much attention has been paid to aims and objectives, the content of the curriculum, teaching methods, assessment and educational strategies such as problem-based learning, integration and community-based learning. The conceptual model illustrates how this qualitative element of the learning needs analysis fed into the development of the core competency framework (Fig. 2).

Oncology HCPs reported feeling comfortable in their professions and specialties but showed a lack of understanding on the roles of other HCPs, thereby failing to offer an inter-speciality approach to people affected by cancer. This separation of professions has been caused by the separate education and training they receive in their disciplines with little opportunity to work together out of the day-to-day clinical setting [15, 16].

It was clear that participants were eager to understand better the role of other HCPs. They recognised the value of IPL and IST in improving communication and care to patient and family. Many had difficulty in understanding the concept of IST. The main goal of IPL is to support HCPs from different professions develop a common understanding of how to work together collaboratively [17]. While there is evidence of how to approach IST [18], most papers on IST in oncology fail to give a definition of the meaning of an IST programme [17, 19]. In order to build an IST curriculum, an explanation of IPL and IST should be explicit at the beginning of the competency framework and in the development of the full curriculum. Inter-speciality training for the pilot will include clinical rotations and programmes of education within a profession, for example medicine and nursing where specialists learn together.

Participants also found that working together gave better sense to their work and improved their confidence and job satisfaction. This has been already identified in previous research done with participants of IPL and IST. Costello et al. [16] demonstrated that students felt more comfortable working as a team and felt more capable to improve patients’ outcomes after IPL and IST. It is essential that, to care for people affected by cancer, HCPs work together to provide holistic care that is seamless and of high quality. Currently, this style of pedagogical learning is sparse across the EU particularly for postgraduates. In cancer care, IPL and IST have been used in different environments generally improving knowledge, communication and teamwork [20, 21].

Respondents also identified some barriers and challenges regarding the lack of access to resources and the reluctance to change a hierarchical culture where decisions are not shared. Similar results have been found in a study exploring radiation oncology professionals’ attitudes to IST [17]. Evaluating these barriers and challenges can help to develop a stronger framework with opportunities on how to overcome them.

This theme will be explicit at the beginning of the curriculum and competency framework and woven throughout the document. It is essential that, to care for people affected by cancer, HCPs require to work together. Currently, this style of pedagogical learning is patchy and unevenly distributed across the EU.

### Strengths and Limitations

This study has been done with cancer care specialist professionals and patient advocates from all across Europe, thereby reflecting a broad understanding of the situation.

As participants were constituent societies of ECO and volunteered to take part, they may already be aware of the differences across Europe. As they come from a range of backgrounds with a European perspective, the results of this qualitative survey have special relevance in the European context.

A limitation that was abundantly clear at the beginning was the confusion on what IST is and the distinction between IPL and IST concepts.

As all participants were active in European speciality societies, the findings may not be representative of all HCPs in the oncology field. This may be a limitation in the qualitative rigour of the study [9]. Future research could benefit from including the perspective of HCPs working in the community.

## Conclusion

This research study confirms that there is a lack of understanding on the concept of IST and minimal educational preparations are in place for HCPs to utilise this approach. The development of an IST in oncology will require collaboration of the INTERACT-EUROPE programme with national postgraduate training programmes.

HCPs working within cancer care welcome resources to enable them to provide the best possible care for people affected by cancer. It is acknowledged that a high standard of cancer education is required for the delivery of quality patient care. Participants in this study recognised the need to be prepared both educationally and practically in order to acquire the knowledge and skills to care efficiently and effectively for this patient group and their families.

Developments in cancer care are rapidly changing healthcare services. There is a need to develop inter-speciality competence and deepen knowledge across the cancer trajectory, from cancer prevention, screening and early detection through diagnostics, treatment, rehabilitation, survivorship, supportive and palliative care to end-of-life care. There is an opportunity to learn from other HCPs to improve the overall care for people affected by cancer and their families. Results of this study will inform the design and development of an IST curriculum and framework for cancer professionals.
