# Correction to: Like Frying Multiple Eggs in One Pan: a Qualitative Study Exploring the Understanding of Inter-speciality Training in Cancer Care

**DOI:** 10.1007/s13187-023-02299-4

**Published:** 2023-04-14

**Authors:** W. McInally, K. Benstead, A. Brandl, N. Dodlek, J. De Munter, C. Gasparotto, J. Grau-Eriksen, R. G. Kelly, C. Lecoq, N. O’Higgins, K. Oliver, M. Popovics, I. Rollo, V. Sulosaari, Celia Díez de los Ríos de la Serna

**Affiliations:** 1 The Open University, Milton Keynes, UK; 2 Gloucestershire Hospitals NHS Foundation Trust, Gloucester, UK; 3 Charite Berlin, Berlin, Germany; 4 Cyprus University of Technology, Limassol, Cyprus; 5 Ghent University Hospital, Brussels, Belgium; 6 European Society for Radiotherapy and Oncology, Brussels, Belgium; 7 Aarhus University Hospital, Aarhus, Denmark; 8 European Oncology Nursing Society, Brussels, Belgium; 9 European Society of Surgical Oncology, Brussels, Belgium; 10 University College Dublin, Dublin, Ireland; 11 European Cancer Organisation, Brussels, Belgium; 12 Turku University of Applied Sciences, Turku, Finland; 13 University of Barcelona, Barcelona, Spain

**Correction to: Journal of Cancer Education**

**https://doi.org/10.1007/s13187-023-02285-w**

The original version of this article unfortunately contained a mistake.

The author name Celia Díez de los Ríos de la Serna is now corrected in the author group.

The original article has been corrected.

